# Vitamin B12 Deficiency and Hemoglobin H Disease Early Misdiagnosed as Thrombotic Thrombocytopenic Purpura: A Series of Unfortunate Events

**DOI:** 10.1155/2015/478151

**Published:** 2015-11-02

**Authors:** Panagiotis Andreadis, Stamatia Theodoridou, Marily Pasakiotou, Stergios Arapoglou, Eleni Gigi, Evaggelia Vetsiou, Efthymia Vlachaki

**Affiliations:** ^1^Second Department of Internal Medicine, Aristotle University, Hippokration Hospital, 54642 Thessaloniki, Greece; ^2^Thalassemia Unit, Hippokration Hospital, 54642 Thessaloniki, Greece; ^3^Intensive Care Unit, Hippokration Hospital, 54642 Thessaloniki, Greece; ^4^Fifth Surgical Clinic, Aristotle University, Hippokration Hospital, 54642 Thessaloniki, Greece

## Abstract

We herein would like to report an interesting case of a patient who presented with anemia and thrombocytopenia combined with high serum Lactic Dehydrogenase where Thrombotic Thrombocytopenic Purpura was originally considered. As indicated a central venous catheter was inserted in his subclavian vein which led to mediastinal hematoma and finally intubation and Intensive Care Unit (ICU) hospitalization. After further examination patient was finally diagnosed with B12 deficiency in a setting of H hemoglobinopathy. There have been previous reports where pernicious anemia was originally diagnosed and treated as Thrombotic Thrombocytopenic Purpura but there has been none to our knowledge that was implicated with hemothorax and ICU hospitalization or correlated with thalassemia and we discuss the significance of accurate diagnosis in order to avoid adverse reactions and therapy implications.

## 1. Introduction

Thrombotic Thrombocytopenic Purpura (TTP) is a rare life-threatening blood disorder that is characterized by a pentad of clinical findings including microangiopathic hemolytic anemia (MAHA), thrombocytopenia, fever, renal dysfunction, and neurologic manifestations [1, 2]. Most of the time, clinical doctors suspect TTP in cases where patients present with thrombocytopenia, elevated Lactic Dehydrogenase (LDH), and schistocytes in peripheral blood smear examination though it is worth mentioning that schistocytes may be absent at the beginning of the disease. Upon serious suspicion of TTP, therapy should be initiated with exchange plasma transfusion [3] that requires insertion of central venous catheter (CVC) [4].

## 2. Case Report 

A 58-year-old male patient presented to the Emergency Department complaining of five-day history of worsening fatigue and deteriorating weakness. His medical history was unremarkable. From the clinical examination he was in relative good condition, except presenting with pallor. His vitals were normal and further clinical examination revealed hepatomegaly and splenomegaly (both 5 cm under the costal arch). Laboratory workup (Table 1) was noteworthy: severe normocytic normochromic anemia with a hematocrit (Ht) of 11% and hemoglobin (Hb) of 3.8 gr/dL, Mean Corpuscular Volume (MCV) 84.5 fL, and increased Red Blood Cell Distribution Width (RDW) 20%. Platelets (PLTs) count was 130000/mL while white blood cells (WBCs) count was normal. The biochemistry panel had significant findings such as extreme elevation of LDH above 3700 IU/L. Reticulocyte (RC) count was 0.1%. His clotting assay revealed slight elevation of International Normalized Ratio (INR) (1.12) and borderline fibrinogen of 155 mg/dL.

Patient was admitted to our clinic for further investigation and treatment. He was transfused with four Red Blood Cells (RBCs). During series of lab work examination, a drop in the patient's PLT number was observed (55000/mL). Peripheral blood smear examination during admission revealed no schistocytes. Despite of no such finding at the time, differential diagnosis included TTP, Disseminated Intravascular Coagulation (DIC), and intermediate thalassemia. Since TTP was considered as a possible diagnosis, the insertion of a CVC was mandatory.

After explaining the possible implications of such procedure and written consent, patient underwent surgical placement of central venous catheter on his subclavian vein. The cannulation was achieved by “blind” anatomical landmark method, as in all cases in our hospital. Minutes after the cannulation attempt, patient suddenly presented with perspiration, tachycardia, and pallor. Soon afterwords he lost his consciousness. After regaining his level of consciousness, patient started complaining about pain on his right hemithorax where no respiratory sounds could be auscultated and oxygen saturation dropped rapidly. Patient was intubated due to respiratory failure and hemodynamic instability. He went in cardiopulmonary arrest and was successfully resuscitated and later transferred to the ICU. A thoracostomy tube and Bullau tube were placed and blood output of 2400 mL was observed. Postprocedure chest radiograph showed large mediastinal hematoma that occupied the right hemithorax. Thoracoscopy revealed multiple bleeding sites. Patient received transfusions with RBCs and Fresh Frozen Plasma (FFP) and his condition stabilized.

**Figure 1:**
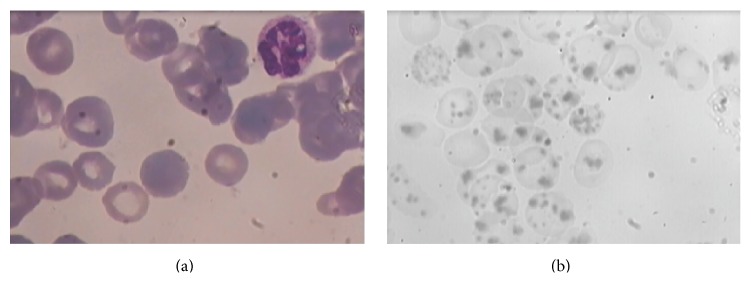
(a) Peripheral blood smear. A hypersegmented neutrophil is found on the right upper corner of the picture. (b) Peripheral blood smear depicting abundant Heinz bodies in our patient with hemoglobin H disease.

Reevaluation of the patient's peripheral blood smear by a specialized hematologist revealed hypersegmented neutrophils, basophilic stippling, anisocytosis and poikilocytosis, reticulocytopenia, and complete absence of schistocytes (Figure 1(a)). Patient underwent bone marrow aspiration and findings included eighty percent erythroblast reaction (erythroid hyperplasia) and few megakaryocytes. Due to high clinical suspicion of megaloblastic anemia, B12 serum levels were ordered and B12 deficiency was confirmed (B12 serum levels 155 pg/mL after 4 RBC and 3 FFP units transfusion). Patient received treatment with oral folic acid (5 mg/d) and intramuscular (i.m.) cobalamin (1000 *μ*g/d). Patient's Ht and PLT count were stable and rising. After five days of hospitalization (stay) in the ICU, he was transferred to the surgery department and later on was discharged with instructions for oral folic acid supplement and i.m. cobalamin once/week for a month and reexamination as an outpatient in our hematology clinic.

During follow-up, patient was feeling well and healthy. Ht was above 35% and PLT count was between normal ranges (Table 1). Forty-five days after the last blood transfusion, in order to allow apoptosis of the transfused RBCs, peripheral blood smear examination revealed abundant Heinz bodies (Figure 1(b)) and hemoglobin electrophoresis revealed hemoglobin H disease. Patient underwent endoscopy and gastric mucosal biopsy revealed atrophic gastritis. He is now healthy continuing treatment with vitamin B12 and folic acid.

## 3. Discussion 

TTP is a rare blood disorder that pathophysiologically arises from inappropriate clot formation in small blood vessels and results in microangiopathic hemolytic anemia (MAHA) and thrombocytopenia whereas in its course it can present with purpura, neurologic disorders, fever, and renal dysfunction (TTP pentad of clinical findings). The term Thrombotic Microangiopathy (TMA) has been attributed to TTP together with other clinical entities such as hemolytic uremic syndrome (HUS), Disseminated Intravascular Coagulation (DIC), and Antiphospholipid Syndrome (APS) but it may also occur in cases of malignant hypertension, systematic lupus erythematosus, preeclampsia, and others [4]. TTP has been associated with infection, autoimmune diseases, and idiopathic deficiency of ADAMS13 protein. A clinical setting that includes thrombocytopenia, anemia with accompanying elevation in LDH, and indirect bilirubin with negative Coombs Test should give rise to suspicion of TTP. Despite the fact that schistocytes are an expected finding in a setting of MAHA, they might be absent at the beginning of the disease. There have also been reported cases where in the absence of schistocytes TTP was suspected and successfully treated with plasmapheresis [5, 6].

Differential diagnosis demands tiering of possible diagnosis according to severity and possibility. TTP is potentially life-threatening and upon clinical suspicion treatment measures should be taken, in this case being plasma exchange transfusion (PEX). Prior to PEX, mortality was nearly 90%. Furthermore, plasma transfusion and PEX increased survival to 58 and 71% accordingly [7].

Despite studies about the use of peripheral venous access in PEX [8, 9], insertion of a central venous catheter (CVC) is highly indicated and is common practice [10]. Patient must undergo this surgical procedure after written consent and after having been informed about the necessity of the latter and possible implications that include pneumothorax, hemorrhage, deep vein thrombosis, central-line associated infections, and arrhythmias [10, 11]. The placement of CVC may be accomplished using ultrasound guidance or using “blind” anatomical landmark method. There have been meta-analyses that show superiority of the real-time 2D ultrasound assisted-guided CVC placement over the anatomical landmark method [12].

Megaloblastic anemia is a result of defective DNA synthesis and relatively unaffected RNA and cytoplasmic components synthesis. The two latter conditions lead to a delay in cell proliferation and to the formation of “trademark” large RBCs with an arrest in nuclear maturation. Bone marrow findings typically include large erythroblasts (megaloblasts) and hypercellularity [13]. Main morphological feature is macrocytosis which leads to classification in macrocytic anemias and in terms of laboratory findings is correlated with increased MCV. Main causes are nutritional deficiencies such as vitamin B12 (e.g., pernicious anemia) and folic acid, while other causes may include drugs, hypothyroidism, liver disease and alcohol abuse, hemolytic anemia, and myelodysplastic syndromes [14]. Despite the fact that the disease is mostly related with disorders of RBCs, in advanced stages it can affect all cell lines (WBCs, PLTs) and eventually leads to pancytopenia.

Hemoglobin H disease is a form of *α*-thalassemia syndrome that is mostly observed in areas of the Middle East, Mediterranean, and the tribal belt of India. The disease is characterized by variable degree of *α*-globin chain deficit due to mutations-deletions affecting *α*-globin genes [15]. Due to insufficient production of *α*-globin chain, *β*-globin chains create precipitates and form tetramers (*β*_4_), known as hemoglobin H (Hb H). In many cases, patients remain asymptomatic and transfusion-free. Diagnosis may not be set until the first episode of acute hemolytic crisis [15, 16]. As expected common findings are those of jaundice, indirect hyperbilirubinemia, LDH elevation, and high RC count. Peripheral blood smear may show target cells, microcytosis, hypochromia, and anisopoikilocytosis. Hepatosplenomegaly is a common finding in patients with Hb H disease [17]. Vitamin B12 and folic acid deficiencies are very common and may develop in cases of hemoglobinopathies due to increased erythropoiesis. In such cases, patients do not exhibit the expected microcytosis due to lack of factors leading to macrocytosis, though an elevated Red Blood Cell Distribution Width (RDW) is expected. Therefore anemia in Hb H disease is primarily a result of hemolysis (due to oxidative stress, infection, or hypersplenism) and secondly a result of ineffective erythropoiesis (due to lack or consumption of necessary cofactors) [15].

## 4. Conclusions

There have been few cases reported to literature where vitamin B12 deficiency has been early misdiagnosed as TTP. Patients with pernicious anemia presented with pseudo-microangiopathic hemolytic anemia that was unsuccessfully treated with plasma exchange transfusions until the establishment of B12 deficiency diagnosis [18–22]. The clinical situation that we describe is unique for two reasons. First of all there have not been previously any reports of pseudo-TMAs in patients with combined B12 deficiency and Hb H disease and second of all because early resuscitation measures, that is, central venous catheter insertion, led to severe complications that required ICU hospitalization and intubation. Clinicians should be aware that despite the fact that nutrients deficiency is mostly considered a clinical entity that requires outpatient management and follow-up, patients may also present to the Emergency Department in urgent condition. Doctors should also bear in mind that hemoglobin H disease and other thalassemias may be an underlying, undiagnosed condition leading to anemia, especially in high endemic areas and where nutrients deficiency may further implicate the course of the disease.

## Figures and Tables

**Table 1:** Patient's blood results: (1) during admission, (2) after the transfusion of 4 packed RBCs units, (3) after the CVC insertion, hemothorax and intubation, (4) at discharge after having received therapy for B12 deficiency, (5) and finally during reevaluation as an outpatient.

	(1) Admission	(2) After transfusion	(3) After CVC placement	(4) Discharge	(5) Reevaluation
Hematocrit (%)	10.8	23.17	18.8	28.8	35.2
Hemoglobin (gr/dL)	3.1	7.99	6.36	9.2	10.9
Red Blood Cells (10^6^/*μ*L)	1.3	2.74	2.29	3.54	5.06
Mean Corpuscular Volume (MCV) (fL)	84.5	82.31	85.1	81.3	69.6
Mean corpuscular hemoglobin (MCH) (pg)	29.1	28.24	29.3	25.9	21.5
Mean corpuscular hemoglobin concentration (MCHC) (g/dL)	33.1	34.3	33.8	31.9	30.9
Reticulocytes (%)	0.1				3
White blood cells (10^3^/*μ*L)	8.50	3.9	9.5	6.34	10.81
Neutrophils (%)	90.2	95.4	95.4	61	66
Lymphocytes (%)	8.4	3.8	3.8	16.9	24.8
Monocytes (%)	0.6	0.6	0.6	8.4	5.6
Eosinophils (%)	0.1	0.06	0.06	12.9	3.7
Platelets (10^3^/*μ*L)	130	55	25	359	252

Prothrombin time, PT (Sec)	12.9	12.9	15	13.2	11.2
International Normalized Ratio (INR)	1.12	1.14	1.33	1.17	0.99
Activated partial thromboplastin time (APTT) (sec)	33.6	34.8	30.9	30.6	27.8
Fibrinogen (mg/dL)	155	160	139	327	338.4

Lactic dehydrogenase (LDH) (U/L)	3700	2100	1769	182	252
Coombs Test	Negative	Negative			
